# Natural Antioxidant Resveratrol Suppresses Uterine Fibroid Cell Growth and Extracellular Matrix Formation In Vitro and In Vivo

**DOI:** 10.3390/antiox8040099

**Published:** 2019-04-12

**Authors:** Hsin-Yuan Chen, Po-Han Lin, Yin-Hwa Shih, Kei-Lee Wang, Yong-Han Hong, Tzong-Ming Shieh, Tsui-Chin Huang, Shih-Min Hsia

**Affiliations:** 1 School of Nutrition and Health Sciences, College of Nutrition, Taipei Medical University, Taipei 11031, Taiwan; hsin246@gmail.com (H.-Y.C.); phlin@tmu.edu.tw (P.-H.L.); 2 Department of Healthcare Administration, Asia University, Taichung 41354, Taiwan; evashih@asia.edu.tw; 3 Department of Nursing, Ching Kuo Institute of Managemnet and Health, Keelung 20301, Taiwan; kellywang@tmu.edu.tw; 4 Department of Nutrition, I-Shou University, Kaohsiung 84001, Taiwan; yonghan@isu.edu.tw; 5 Department of Dental Hygiene, College of Health Care, China Medical University, Taichung 40402, Taiwan; tmshieh@mail.cmu.edu.tw; 6 PhD Program for Cancer Biology and Drug Discovery, College of Medical Science and Technology, Taipei Medical University and Academia Sinica, Taipei 11031, Taiwan; 7 Graduate Institute of Metabolism and Obesity Sciences, College of Nutrition, Taipei Medical University, Taipei 11031, Taiwan; 8 School of Food and Safety, Taipei Medical University, Taipei 11031, Taiwan; 9 Nutrition Research Center, Taipei Medical University Hospital, Taipei 11031, Taiwan

**Keywords:** uterine fibroids, resveratrol, extracellular matrix, ELT-3-LUC xenograft model

## Abstract

Resveratrol (RSV) is a polyphenolic phytoalexin found in peanuts, grapes, and other plants. Uterine fibroids (UF) are benign growths that are enriched in extracellular matrix (ECM) proteins. In this study, we aimed to investigate the effects of RSV on UF using in vivo and in vitro approaches. In mouse xenograft models, tumors were implanted through the subcutaneous injection of Eker rat-derived uterine leiomyoma cells transfected with luciferase (ELT-3-LUC) in five-week-old female nude (Foxn1^nu^) mice. When the tumors reached a size of 50–100 mm^3^, the mice were randomly assigned to intraperitoneal treatment with RSV (10 mg·kg^−1^) or vehicle control (dimethyl sulfoxide). Tumor tissues were assayed using an immunohistochemistry analysis. We also used primary human leiomyoma cells as in vitro models. Cell viability was determined using the sodium bicarbonate and 3-(4,5-dimethylthiazol-2-yl)-2,5-diphenyltetrazolium bromide (MTT) assay. The protein expression was assayed using Western blot analysis. The messenger ribonucleic acid (mRNA) expression was assayed using quantitative reverse transcription–polymerase chain reaction (qRT–PCR). Cell apoptosis was assayed using Annexin V-fluorescein isothiocyanate (FITC) and propidium iodide (PI) and Hoechst 33342 staining. RSV significantly suppressed tumor growth in vivo and decreased the proportion of cells showing expression of proliferating cell nuclear antigen (PCNA) and α-smooth muscle actin (α-SMA). In addition, RSV decreased the protein expression of PCNA, fibronectin, and upregulated the ratio of Bax (Bcl-2-associated X) and Bcl-2 (B-cell lymphoma/leukemia 2) in vivo. Furthermore, RSV reduced leiomyoma cell viability, and decreased the mRNA levels of fibronectin and the protein expression of collagen type 1 (COL1A1) and α-SMA (ECM protein marker), as well as reducing the levels of β-catenin protein. RSV induced apoptosis and cell cycle arrest at sub-G1 phase. Our findings indicated the inhibitory effects of RSV on the ELT-3-LUC xenograft model and indicated that RSV reduced ECM-related protein expression in primary human leiomyoma cells, demonstrating its potential as an anti-fibrotic therapy for UF.

## 1. Introduction

Benign uterine fibroids (UF), also known as myomas or leiomyomas, are the most common neoplasm of the uterus and occur in up to 77% of women by the onset of menopause in the United States [1,2]. Women with UF usually suffer from a reduced quality of life due to symptoms such as abnormal uterine bleeding, pelvic pain, frequent urination, and infertility [3,4]. Although the etiology remains unclear, genetic factors, cytokines, growth factors, steroid hormones (estrogens and progestogens) and/or their receptors, and excessive production of extracellular matrix (ECM) have been reported to play a pivotal role in the development of UF [4]. In general, the degradation of ECM is precisely regulated under normal physiological conditions, however, abnormal ECM metabolism is involved in pathogenesis of UF [5]. The major ECM components of UF include fibronectin, collagens, and proteoglycans such as biglycan and fibromodulin [6,7].

Most therapeutic treatments provide only temporary or partial relief from UF, and are not successful in every patient [8]. In comparison, hysterectomy is considered as the only option and the fastest treatment to reduce pain from UF, especially for women with uterine fibroids but a lack of cognition [9]. However, women who undergo hysterectomies encounter a number of problems, such as pelvic floor disorders, early menopause, and sexual dysfunction; these postoperative complications can be relieved using conventional medical treatment, but the cost related to uterine fibroids is considerable [9]. In recent years, more research has been undertaken to identify natural extracts as adjuncts to chemotherapy. In particular, dietary polyphenols, such as epigallocatechin gallate (EGCG) [10], green tea extract [11], and strawberry extract [12], have been shown to have anti-leiomyoma activities.

Resveratrol (RSV; trans-3,5,4′-trihydroxystilbene) is a natural polyphenolic compound belonging to the stilbene group. RSV is present in several plants [13], including blueberries [14], peanuts [15], and grapes [16], as well as in grape related products, such as wine [17]. In general, fresh grape skins contain 50–100 mg·g^−1^ resveratrol [18,19]. RSV is a potent antioxidant [20] with anti-inflammatory [21], anti-proliferative [22], and anti-adipogenic [23] effects on several cancer cells, including breast [24] and prostate cancers [25], and it might provide a potential treatment for dysmenorrhea [26]. Recently, some studies have showed that RSV can reduce tissue fibrogenesis in chronic kidney diseases [27]. In addition, we have previously shown that RSV inhibits leiomyoma cell proliferation, induces apoptosis, promotes cell cycle arrest, and regulates messenger ribonucleic acid (mRNA) and protein expression of ECM-associated proteins in vitro [28]. These data support the potential of RSV as an alternative therapeutic treatment for UF. However, its effects on leiomyoma growth in vivo remain unclear. Therefore, the present study investigated the effects of RSV on UF growth in a mouse xenograft model in vivo.

## 2. Materials and Methods

### 2.1. Reagents and Antibodies

Dulbecco’s Modified Eagle Medium/Nutrient Mixture F-12 (DMEM/F12), antibiotic-antimycotic solution (100×), and 0.05% trypsin-ethylenediaminetetraacetic acid (EDTA, 1×) were purchased from CAISSON Labs (Smithfield, UT, USA). Fetal bovine serum (FBS), trypan blue, NucBlue™ Live ReadyProbes™ Reagent, a bicinchoninic acid protein assay kit, and enhanced chemiluminescence reagents were purchased from Thermo Fisher Scientific (Waltham, MA, USA). The Annexin V-fluorescein isothiocyanate (FITC) apoptosis detection kit I was purchased from Becton Dickinson (BD) Biosciences (San Jose, CA, USA). Bovine serum albumin (BSA) was purchased from BioShop (Burlington, Canada). Protease and phosphatase inhibitor cocktail tablets were purchased from Roche (Basel, Switzerland). Sodium bicarbonate, 3-(4,5-dimethylthiazol-2-yl)-2,5-diphenyltetrazolium bromide (MTT), propidium iodide (PI), and dimethyl sulfoxide (DMSO) were purchased from Sigma-Aldrich (Louis, MO, USA). VivoGlo™ Luciferin (in vivo grade) was purchased from Promega (Fitchburg, WI, USA). Matrigel^®^ basement membrane matrix was purchased from Corning (Corning, NY, USA). Zoletil^®^ 50 was purchased from Virbac (Carros, France). Rompun^®^ 20 (xylazine hydrochloride) was purchased from Bayer (Pittsburgh, PA, USA). The following antibodies were used in this study: anti-proliferating cell nuclear antigen (PCNA), anti-Bax, anti-Bcl-2 and anti-β-catenin (Cell Signal Technology, Danvers, MA, USA), anti-β-actin and anti-α-SMA, anti-vimentin, anti-collagen type 1 (COL1A1) (GeneTex, Irvine, CA, USA), fibronectin, and goat anti-rabbit IgG and anti-mouse IgG antibodies (Abcam, Cambridge, MA, USA).

### 2.2. Preparation of RSV

RSV (C_14_H_12_O_3_, chemical abstracts service number: 501-36-0) was purchased from ECHO CHEMICAL Co., Ltd. (purity >99%, Miaoli, Taiwan). A stock solution of 100 mM was prepared in DMSO, aliquoted, and then stored at −20 °C until use. For in vitro experiments, the final concentrations of RSV were prepared by diluting the stock with cell culture medium. The control cells were treated with vehicle (0.1% DMSO).

### 2.3. Cell Culture

The Eker rat-derived uterine leiomyoma (ELT-3) cell lines were kindly provided by Lin-Hung Wei (Department of Oncology, National Taiwan University Hospital, Taipei, Taiwan). ELT-3 cells transfected with luciferase reporter genes (ELT-3-LUC) were previously established in our laboratory. In addition, the primary cultures of human leiomyoma cells were isolated from uterine leiomyoma tumor tissue specimens, which were collected from women (30–40 years of age, *n* = 6) undergoing myomectomy at the Department of Oncology, National Taiwan University Hospital (Taipei, Taiwan). According to a previous study all the human tissue specimens were approved by the Institutional Review Board and Ethics Committee of the National Taiwan University Hospital (permit number: 201210072RIC). The process of purification of the leiomyoma cells was as described previously [29], and leiomyoma cells from passages 2–7 were used in this study. Both ELT-3-LUC and leiomyoma cells were cultured in DMEM/F12 containing 10% FBS, 1% antibiotics [10,000 units·mL^−1^ penicillin, 10,000 μg·mL^−1^ streptomycin, and 25 μg·mL^−1^ amphotericin with 8.5 g·L^−1^ NaCl], and 0.6 mg·mL^−1^ Geneticin^®^ G418 Sulfate (Thermo Fisher Scientific, Waltham, MA, USA; ELT-3-LUC only); both cell lines were incubated at 37 ℃ with 5% CO_2_.

### 2.4. Tumor Xenograft in Nude (Foxn1^nu^) Mice

Five-week-old female nude (Foxn1^nu^) mice (BioLASCO, Taipei, Taiwan) were housed under a 12 h light/12 h dark cycle in a pathogen-free environment, with ad libitum access to food and water. Tumors were implanted through the subcutaneous (s.c.) injection of ELT-3-LUC cells (1 × 10^6^ cells suspended in 0.1 mL phosphate-buffered saline (PBS)/Matrigel solution for each mouse) into the right flank of the mice. After the tumors reached a size of 50–100 mm^3^ (approximately 1 month), the mice were randomly assigned to two groups (*n* = 5): one group received an intraperitoneal (i.p.) injection of RSV (10 mg·kg^−1^; treatment group), and the other group received a vehicle (DMSO; control group) twice a week for 4 weeks. The tumor volume was measured using calipers and calculated as L × W^2^ × 0.52, where L is the length and W is the width. The tumor volumes and body weights were recorded until the animals were sacrificed by an i.p. injection of anesthetic mixtures [1 mL zoletil (Virbac, Carros, France) + 1 mL rompun (Bayer, Pittsburgh, PA, USA)]. Every week, the mice were administered an i.p. injection of luciferin (150 mg·kg^−1^ body weight) and detected using a non-invasive in vivo imaging system (IVIS). At the end of the experiment, the tumor tissues were stained with hematoxylin and eosin (H&E). All the animal studies were conducted according to the protocols approved by the Institutional Animal Care and Use Committee (IACUC) of Taipei Medical University (IACUC approval no. 2015–0115).

### 2.5. Immunohistochemistry Analysis

To observe the localization of specific proteins, immunohistochemistry analysis was assayed. Tumor tissues were embedded and sliced at a thickness of 2- or 6-μm by the animal experiment center of Taipei Medical University (Taipei, Taiwan). The tissue sections were stained by Bio-Check Laboratories Ltd (Taipei, Taiwan). To analyze the immunohistochemistry slides, five areas were photographed at 40× magnification (center, bottom, top, left, and right regions) using an EVOS^®^ microscope (Thermo Fisher Scientific, Waltham, MA, USA), and the color of the PCNA and α-SMA staining in the tissue sections was observed.

### 2.6. Western Blot Analysis

The lysates of tumor tissues were prepared in ice-cold lysis buffer (50 mmol·L^−1^ Tris (pH 8.0), 100 mmol·L^−1^ NaCl, 0.1% sodium dodecyl sulfate (SDS), 1% NP-40, 0.5 mM EDTA) containing a protease inhibitor cocktail. The proteins (30 μg) were boiled for 5 min, separated using 12% SDS–polyacrylamide gel electrophoresis (SDS–PAGE), and then transferred electrophoretically to Immobilon-P polyvinylidene fluoride (PVDF) membranes (0.22 µm) for 150–180 min at 280 mA and 250 V. Then, the membranes were washed three times for 10 min/wash with Tris-buffered saline containing Tween 20 (TBST) buffer, blocked with blocking buffer (5% BSA) for 1 h at 25℃, and incubated for 8 h with primary antibodies (1:1000 in blocking buffer) at 4 ℃. The next day, the membranes were washed three times for 10 min/wash with a TBST buffer, incubated for 1 h in a blocking buffer containing goat anti-rabbit or anti-mouse IgG (as appropriate) coupled to alkaline phosphatase (1:10,000), and washed three times with TBST (10 min/wash). Finally, the bands were detected using enhanced chemiluminescence. The densitometric values were normalized to the internal control (β-actin) using Image Lab™ Software Version 5.2 1. (Bio-Rad, Hercules, California, USA).

### 2.7. Cell Viability Assay

The effect of RSV treatment on cell viability was examined using the MTT assay. The human leiomyoma cells were seeded in 96-well plates (2 × 10^3^ cells/well), cultured for 24 h, and treated with various concentrations of RSV in fresh medium containing 1% FBS. The MTT solution (1 mg·mL^−1^) was then added directly to each well (100 μL/well) for 4 h. The absorbance was measured at 570 nm, with a reference wavelength of >630 nm, using a microplate reader (BioTek, Winooski, VT, USA).

### 2.8. Quantitative Real-Time RT–PCR (qRT–PCR)

The total cellular ribonucleic acid (RNA) was extracted from RSV-treated cells with TRIzol™ reagent (Thermo Fisher Scientific, Waltham, MA, USA) followed by Quick-RNATM MiniPrep Plus (Zymo Research, Irvine, CA, USA), and a total of 2 µg RNA was reverse transcribed using a RevertAid H minus first strand cDNA synthesis kit (Thermo Fisher Scientific, Waltham, MA, USA) according to the manufacturer’s instructions. Amplification reactions were performed using the PowerUp™ SYBR™ Green master mix (Thermo Fisher Scientific, Waltham, MA, USA). qRT–PCR analyses were performed using the Applied Biosystems StepOnePlus™ real-time PCR system (Thermo Fisher Scientific, Waltham, MA, USA). Amplification of all genes was performed under the following cycling conditions: denaturation at 95 ℃ for 10 min, followed by 40 cycles for 15 s at 95 ℃ and 30 s at 60 ℃. The synthesis of the DNA product of the expected size was confirmed using a melt curve analysis. The comparative threshold cycle (Ct) values of each gene were normalized to Ct values of glyceraldehyde 3-phosphate dehydrogenase (GAPDH, internal control). The primers used for qRT–PCR analysis are listed in Table 1.

### 2.9. Hoechst 33342 Staining

To detect alterations of nuclei morphology of leiomyoma cells after RSV treatment, Hoechst 33342 staining was performed. The leiomyoma cells were seeded in 6 cm^2^ culture dishes (5 × 10^4^ cells) and treated with RSV (10, 50, 100 μM). After 48 h of treatment, the cells were directly stained with 2 drops/mL Hoechst 33342 by incubation for 20 min at room temperature. Images were acquired using a fluorescence microscope.

### 2.10. Apoptosis Analysis

The induction of apoptosis was determined using Annexin V-FITC/PI staining. The leiomyoma cells were seeded in 10 cm^2^ culture dishes (1 × 10^6^ cells) and treated with RSV (10, 50, 100 μM) for 48 h. The cells were stained with Annexin V-FITC and PI by incubation for 15 min at room temperature protected from light. The apoptotic cells were analyzed using BD Accuri™ C6 Plus Flow Cytometer (BD Biosciences, San Jose, CA, USA), and the results were analyzed using the BD Accuri™ C6 Plus software (BD Biosciences, San Jose, CA, USA).

### 2.11. Cell Cycle Analysis

To assess the cell cycle progression, the leiomyoma cells were seeded into 10 cm^2^ culture dishes (1 × 10^6^ cells) and then treated with RSV (10, 50, 100 μM) for 48 h. All the cells were collected, slowly added to 9 mL of 70% cold ethanol, and then stored at −20 ℃ overnight. The cells were washed twice with cold phosphate-buffered saline (PBS), resuspended in 500 µL propidium iodide (PI)/Triton X-100 staining solution (10 mL 0.1% (*v*/*v*) Triton X-100 in PBS containing 2 mg DNAse-free RNAse A and 0.40 mL of 500 µg·mL^−1^ PI), and incubated for 30 min at 20 ℃. The fluorescence was measured using a fluorescence-activated cell-sorting (FACS) Calibur flow cytometer (BD Biosciences, San Jose, CA, USA) and the cell cycle distribution was analyzed using the CellQuest software program (BD Biosciences, San Jose, CA, USA).

### 2.12. Statistical Analysis

The data are presented as the mean ± standard deviation (SD), and the differences between the means were analyzed using Sigma Plot version 12.5 (SoftHome International, Taipei, Taiwan). For the comparison of the two groups, a Student’s *t*-test was used. The group means were compared using the one-way analysis of variance and Duncan’s multiple-range test. The difference between two means was considered as statistically significant when *p* < 0.05.

## 3. Results

### 3.1. The Inhibitory Effect of RSV on the Growth of UF in Vivo

**Figure 1:**
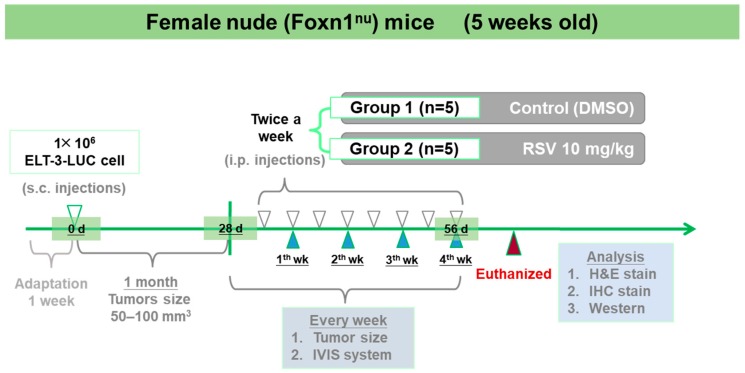
Schematic representation of the treatment plan for the xenograft mouse model. Cultured Eker rat-derived uterine leiomyoma cells transfected with luciferase (ELT-3-LUC) embedded in Dulbecco’s Modified Eagle Medium/Nutrient Mixture F-12 (DMEM/F12)/Matrigel solution were transplanted into the right flank of female nude (Foxn1^nu^) mice. When the tumors reached a size of 50–100 mm^3^ (approximately 1 month), the mice received an intraperitoneal injection of either resveratrol (RSV; 10 mg·kg^−1^) or vehicle control (dimethyl sulfoxide; DMSO) twice a week for 1 month. nude (Foxn1^nu^) mice: nude mice with a spontaneous deletion in the *FOXN1* gene; IVIS: non-invasive in vivo imaging system; H&E: hematoxylin and eosin; IHC: immunohistochemical.

**Figure 2:**
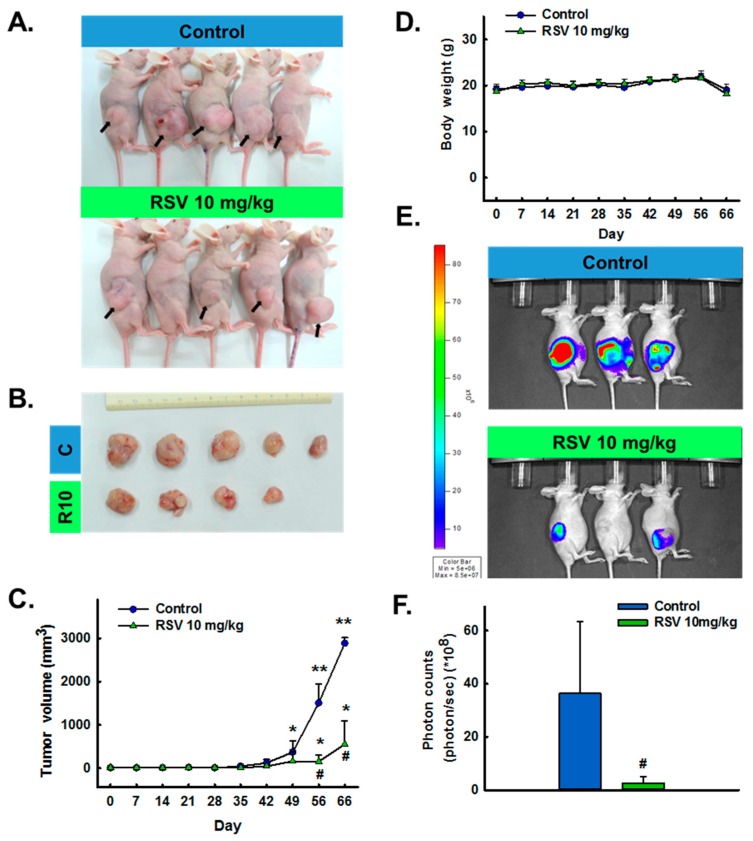
Effect of resveratrol (RSV) on tumor xenograft growth. (**A**) Morphology and (**B**) size of tumors isolated from the sacrificed mice in each group are shown at day 66, the end-point of the treatment. (**C**) Tumor volumes and (**D**) body weights of nude (Foxn1^nu^) mice. (**E**) Total photon flux from imaging on day 56 after xenografting. (**F**) All luciferase images were normalized to the same photon saturation scale. Data are presented as the mean ± SD (*n* = 5 or 3); * *p* < 0.05 and ** *p* < 0.001 vs. day 0; # *p* < 0.05 vs. control.

As shown in Figure 1, the treatment group received RSV via i.p. injection twice per week for 4 weeks. During the treatment period, the mouse body weights were measured each time they were injected to investigate the effects of RSV on overall health. IVIS was used to track ELT-3-LUC tumor growth over time in this mouse xenograft model. Unfortunately, due to the individual differences and ELT-3-LUC cell instability, we did not show all the tracking results. From the appearance and size of the tumors, we can initially evaluate the effect of resveratrol. The tumor sizes and volumes were significantly reduced in the treatment group, as compared to the control group (Figure 2A,B). Notably, a significant difference in tumor volume was observed between the vehicle- and RSV-treated groups from day 56 of treatment (Figure 2C). No significant group difference in the mouse body weights was observed (Figure 2D). In addition, IVIS imaging identified a higher bioluminescent signal in the vehicle-treated group than in the RSV group at day 56, although large inter-individual differences were observed (Figure 2E,F). These data demonstrated the potent inhibitory effect of RSV on the growth of UF within a relatively short treatment period.

**Figure 3:**
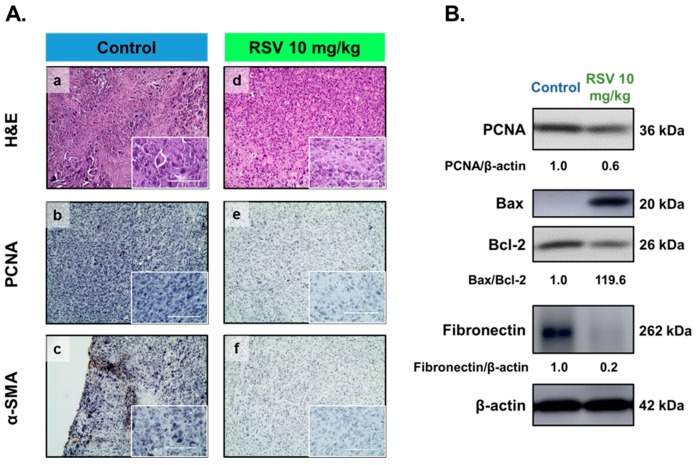
Effect of resveratrol (RSV) treatment on tumor xenografts. (**A**) Eker rat-derived uterine leiomyoma cells transfected with luciferase (ELT-3-LUC) tumors were excised and processed for hematoxylin and eosin (H&E) staining (**a**,**d**) and immunohistochemical (IHC) analysis of proliferating cell nuclear antigen (PCNA) (**b**,**e**) and α-smooth muscle actin (α-SMA) **(c**,**f**); the scale bars represent 100 μm. (**B**) Tumor lysates were separated by sodium dodecyl sulfate polyacrylamide gel electrophoresis and analyzed on Western blotting with an anti-PCNA, fibronectin, Bax and Bcl-2 antibody. β-actin was used as a loading control. The band intensities are expressed as a ratio, relative to the loading control.

To explore the effects of RSV further, immunohistochemical analyses were performed. Compared with the control group, the RSV-treated (10 mg·kg^−1^) group showed a decrease in the proportions of cells that were positive for PCNA (a marker of cell growth, Figure 3A-b,-e) or α-SMA (a smooth muscle marker, Figure 3A-c,-f), as well as hematoxylin and eosin staining (H&E, Figure 3A-a,-d). In addition, Western blot analysis showed that mice treated with RSV showed reduced levels of PCNA and fibronectin in whole tissue extracts (Figure 3B), but enhanced levels of Bax/Bcl-2 (apoptosis-related markers).

### 3.2. Effects of RSV on Leiomyoma Cell Proliferation and Extracellular Matrix (ECM) Accumulation in Vitro

**Figure 4:**
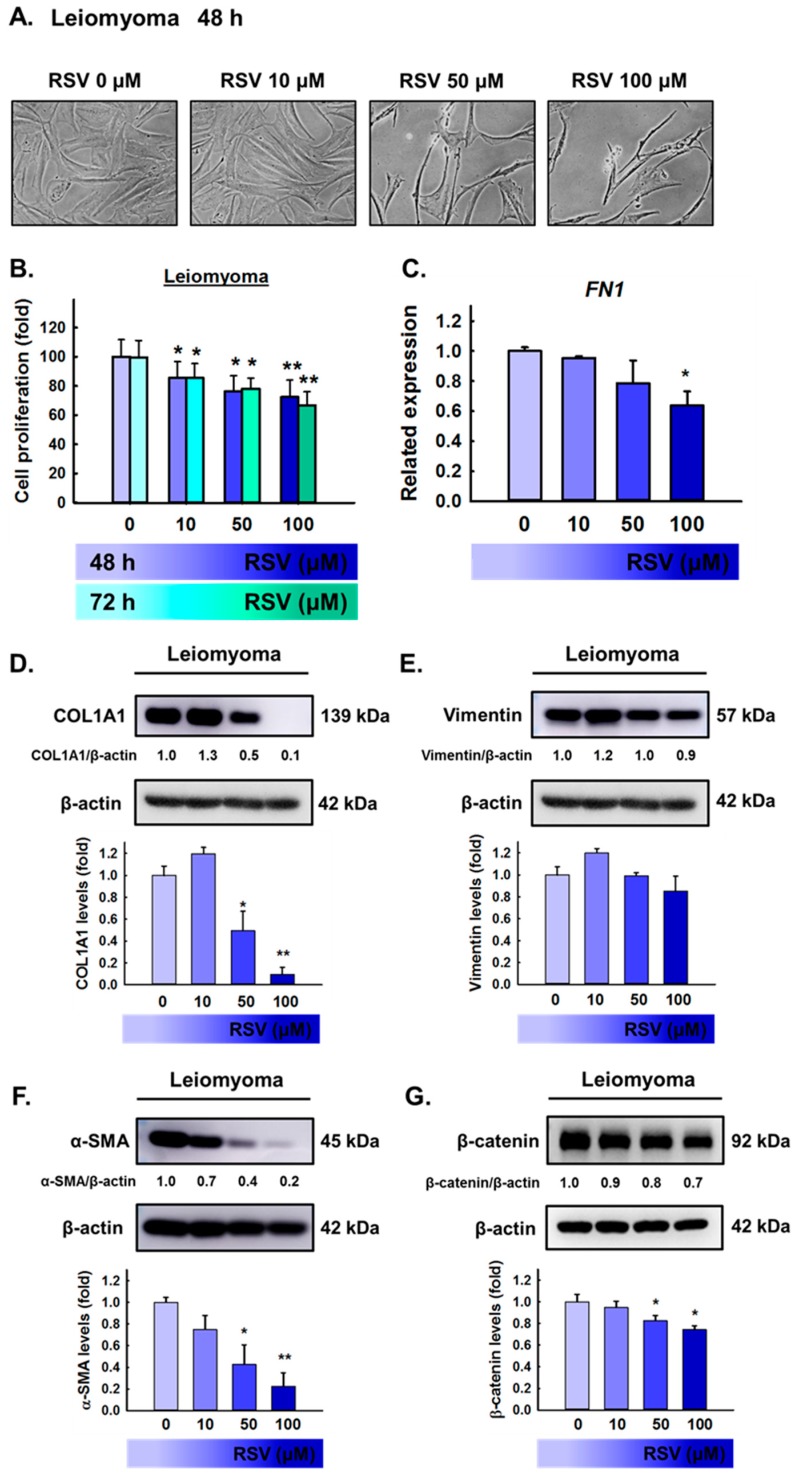
Cytotoxic effects of resveratrol (RSV) on primary human leiomyoma cells. Leiomyoma cells were exposed to either vehicle (dimethyl sulfoxide; DMSO) or RSV (10–100 μM) for 48 h or 72 h. (**A**) Morphology of leiomyoma cells after the indicated treatment (magnification, ×200). (**B**) Cell proliferation was measured using a 3-(4,5-dimethylthiazol-2-yl)-2,5-diphenyltetrazolium bromide (MTT) assay. (**C**) RNA samples were isolated from leiomyoma cells treated with RSV (0–100 μM) and subjected to quantitative reverse transcription–polymerase chain reaction (qRT–PCR) using primers specific for fibronectin (*FN1*). (**D**–**G**) Leiomyoma cell lysates were separated using sodium dodecyl sulfate polyacrylamide gel electrophoresis (SDS–PAGE) and analyzed using Western blot with anti-COL1A1, vimentin, α-SMA, and β-catenin. β-actin was used as a loading control. The values of the band intensity represent the densitometric estimation of each band normalized to β-actin. Protein quantification of COL1A1, vimentin, α-SMA, and β-catenin expression in leiomyoma cells is shown in the bar graph. The results are expressed as the means ± SD of three independent experiments; * *p* < 0.05, ** *p* < 0.001, as compared with the control.

To evaluate whether RSV produced a cytotoxic effect, leiomyoma cells were treated with RSV (10, 50, or 100 μM) for 48 h or 72 h. Cell viability was measured using the MTT assay; the results showed that RSV has significantly reduced the viability of leiomyoma cells (Figure 4B), and narrow cells were observed at 100 μM RSV (Figure 4A). Numerous studies have shown that excessive ECM production is an important factor that cannot be ignored in relation to uterine fibroid growth. To examine the effect of RSV on the expression of ECM in leiomyoma cells, we chose more representative ECM proteins as markers, such as fibronectin, collagen type 1, vimentin, and α-SMA. As shown in Figure 4C, leiomyoma cells exposed to 100 μM RSV showed a significantly lower mRNA expression of *FN1*. In addition, Western blot analysis showed that 100 μM RSV significantly decreased the levels of COL1A1, α-SMA, and β-catenin compared to controls for 48 h (Figure 4D,F,G). These data demonstrate the potent inhibitory effect of RSV on tumor growth and ECM accumulation in leiomyoma cells in vitro.

### 3.3. Effects of RSV on Apoptosis and Cell Cycle Progression of Leiomyoma Cells in Vitro

**Figure 5:**
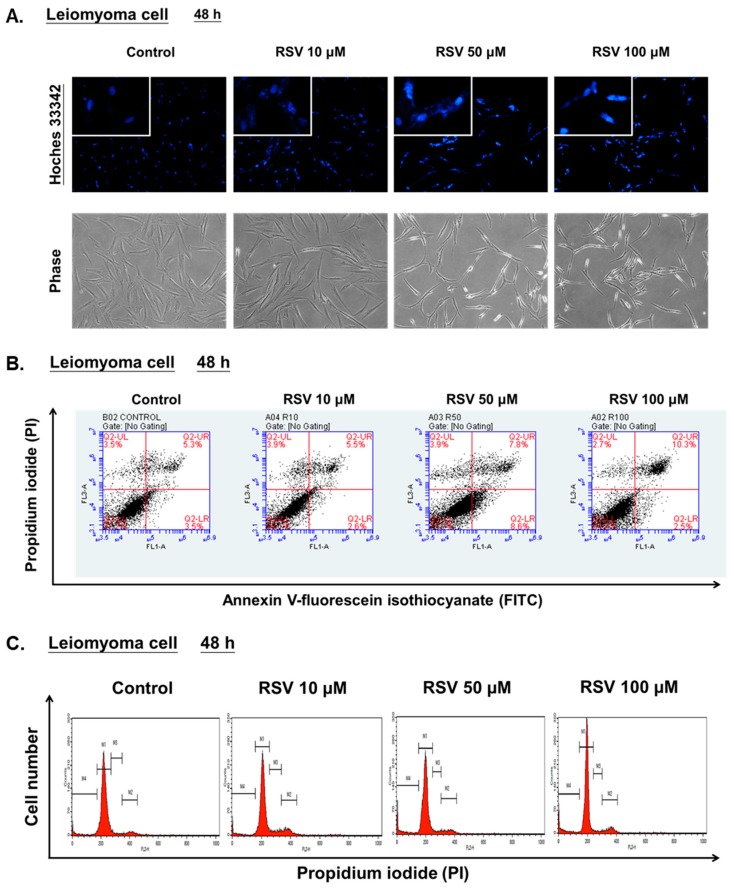
Resveratrol (RSV) induces apoptosis in primary human leiomyoma cells. Leiomyoma cells were exposed to either vehicle (dimethyl sulfoxide; DMSO) or RSV (10, 50, 100 μM) for 48 h. (**A**) Nuclear changes revealed by Hoechst 33342 (×200) and the morphology of leiomyoma cells. (**B**) The cells were harvested and stained with Annexin V-fluorescein isothiocyanate (FITC) and propidium iodide (PI), and cell apoptosis was analyzed using flow cytometry. (**C**) The cells were stained with propidium iodide (PI), and the histograms of cell cycle distribution was analyzed by flow cytometry.

Nuclear condensation and the nuclear morphology changes in leiomyoma cells were examined by using Hoechst 33342 staining at 48 h after RSV treatment. As shown in Figure 5A, leiomyoma cells exposed to 100 μM RSV showed stronger blue fluorescence and an increased number of cells with fragmented and condensed nuclei than the control group. To evaluate whether RSV induced apoptosis, Annexin V-FITC and PI staining were used. The apoptosis rate depended on the percentage of early apoptotic cells (FITC+/PI−) and late apoptotic cells (FITC+/PI+). As shown in Figure 5B, 100 μM RSV increased the percentage of apoptotic cells compared to the controls at 48 h. On the other hand, the fluorescence intensity of the sub-G1 cell fraction also represented an apoptotic cell population. As shown in Figure 5C, 100 μM RSV increased the percentage of sub-G1 cells compared to controls at 48 h. These data demonstrate that RSV has potent pro-apoptosis effects on tumor growth in leiomyoma cells in vitro.

## 4. Discussion

This study identified potentially beneficial inhibitory effects of RSV on UF growth in a mouse xenograft model in vivo, as well as on the proliferation of primary human leiomyoma cells in vitro. RSV exhibits pleiotropic activities in both in vivo and in vitro experimental models; these include anti-proliferation, pro-apoptosis, anti-carcinogenic, and anti-oxidant effects [20,22,24,25]. Each cell line has a different sensitivity to RSV and individual animal models also have different outcomes according to varying experimental conditions. For example, the intraperitoneal (i.p.) injection of RSV at a dose of 25 mg·kg^−1^ body weight reduced the tumor volume of MDA-MB-231 breast cancer cells in a xenograft mice model [30]. In addition, RSV (2.5 and 10 mg·kg^−1^) administered intraperitoneally significantly reduced the tumor volume in mice bearing highly metastatic Lewis lung carcinoma (LLC) tumors [31]. In our study, we found that the i.p. injection of RSV at a dose of 10 mg·kg^−1^ body weight reduced the tumor volume of ELT-3 uterine leiomyoma cells.

In theory, a higher plasma level of RSV could be reached with a high dose of RSV. However, the consumption of a higher dose of RSV did not necessarily result in significantly higher plasma concentrations. According to a previous study, the plasma bioavailability of RSV was approximately 2% after a single-dose consumption [32], which is the result of rapid biotransformation to sulfate as well as the glucuronide conjugates. In addition, RSV was administered intraperitoneally at a concentration of 20 mg·kg^−1^ body weight; as a result, approximately 5 μM resveratrol glucuronide and 13 μM resveratrol sulfate were detected in the serum after 15 min, with concentrations reducing over the next 2 h [33]. Although the bioavailability of RSV is very low, many studies still use a higher than physiologically reasonable concentration for research purposes. For example, Garvin et al. found that 100 μM RSV induced significant morphological changes indicative of apoptosis in MDA-MB-231 breast cancer cells [30], and Wong et al. also found that 100 μM RSV promoted apoptosis by mediating caspase-3/7 activation and induced morphologic changes in cultured ovarian theca-interstitial (T-I) cells [22], these findings both based on the same concentration utilized in our study. However, in order to improve the bioavailability of RSV, Caddeo et al. changed the form of delivery of natural products and found the effectiveness of RSV can be potentiated by a polyphenol vesicular formulation [34].

The use of athymic nude mice is a commonly employed experimental model for cancer treatment [35]. In previous studies, scientific researchers have also used this mouse xenograft model to explore the therapy of leiomyoma [36,37]. For example, a previous in vivo study on nude mice injected subcutaneously with ELT-3 leiomyoma cells showed that EGCG treatment reduced tumor size, as compared to a control treatment (water). In addition, EGCG arrested the growth of ELT-3 cells and decreased leiomyoma size in Eker rat models as early as two weeks after treatment initiation [10]. Our study referred to the same mouse xenograft model and established a similar method, in which ELT-3 uterine leiomyoma cells were inoculated subcutaneously into the right flank of nude (Foxn1^nu^) mice after anesthetizing. In agreement with the results of the study by Zhang et al. [10], our results showed that RSV significantly decreased tumor volume and arrested tumor growth. It is worth mentioning that Suzuk et al. [36] have established a novel and simple mouse xenograft model of human uterine leiomyomas according to this author’s latest study, which will provide us with an ideal experimental model for the discovery of new compounds in the future.

PCNA is a DNA polymerase coenzyme that is closely related to cell proliferative activity because of its involvement in the synthesis of DNA in the nucleus [38]. In a previous study, immunohistochemical (IHC) staining revealed a decreased PCNA expression in Eker rat leiomyomas treated with 1, 25-dihydroxyvitamin D3, as compared to vehicle-treated control rats [39]. Zhang et al. demonstrated that the number of PCNA-positive cells decreased after 4- and 8-week treatments with EGCG, as compared to the number observed in water-treated control animals [10]. Similarly, the present study identified a decrease in the number of PCNA-positive cells and the protein expression of PCNA in mice treated with RSV, as compared to vehicle-treated animals.

Apoptosis is a process of programmed cell death; both Bax (Bcl-2-associated X) and Bcl-2 (B-cell lymphoma/leukemia 2) are markers of apoptosis-regulating proteins. The expression of Bcl-2 results in prolonged cell survival by restricting the activation of caspases. On the other hand, the overexpression of Bax results in accelerated programmed cell death. According to previous studies, the anti-apoptotic mechanism seems to be involved in the development of uterine leiomyoma; several studies have demonstrated that the anti-apoptotic *Bcl-2* gene was significantly over-expressed in uterine leiomyoma compared to homologous myometrium [40], and can even be influenced by the endocrine environment [41]. A previous study demonstrated that the inhibition of anti-apoptotic proteins Bcl-2/Bcl-xL promoted apoptotic cell death [42]. In the current study, we found that RSV enhanced the ratio of Bax and Bcl-2 and speculated that RSV may have induced apoptosis of UF growth in vivo. In addition, a previous study from Baarine et al. [43] found that RSV-treated cells exhibited apoptosis characteristics including nuclear fragmentation and condensation which were identified by Hoechst 33342. In agreement with the results of the study by Baarine et al. [43], our results showed that 100 μM RSV enhanced blue fluorescence and increased the number of cells with fragmented and condensed nuclei in primary human leiomyoma cells in vitro.

Because ECM accumulation is critical for the development of UF [44], it seems that the inhibitory effects of RSV could be mediated by ECM degradation. There are many factors related to ECM; first of all, increased deposition of ECM-associated proteins (fibronectin, collagens) and proteoglycans (biglycan, fibromodulin) is a typical characteristic of UF [6,7]. Myofibroblasts are the ECM-depositing cells active in wound healing, which are retained by UF when fibrotic responses are dysregulated [45]. The activation of myofibroblasts correlates with the expression of α-SMA, which is a key component supporting tissue contraction of ECM [46]. Previous studies have demonstrated that α-SMA is elevated in leiomyoma compared to myometrium [47]. In addition, several studies have reported that β-catenin expression was increased in UF compared to the adjacent myometrium tissue [48], which is associated with proliferation and ECM formation [49]. A recent study has shown that an increase in ECM stiffness triggers upregulation of β-catenin in UF cells [48].

In our previous study, we found that RSV reduced the levels of ECM-associated proteins (fibronectin and collagen type 1) and proteoglycans (fibromodulin and biglycan) in ELT3 cells in vitro [28]. In agreement with the results of these studies, our results showed that 100 μM RSV significantly decreased the protein expression of COL1A1, α-SMA, and β-catenin, as well as the mRNA level of *FN1* (fibronectin) in primary human leiomyoma cells compared to controls in vitro. Furthermore, RSV (10 mg·kg^−1^) reduced the proportion of α-SMA-positive cells and decreased the protein levels of fibronectin in vivo. However, the limitations of the present study are worth mentioning. The underlying mechanisms of RSV on ELT-3-LUC tumor xenografts still need to be elucidated in detail and further exploration of the molecular mechanisms and biological significance of RSV on ECM degradation is warranted.

## 5. Conclusions

**Figure 6:**
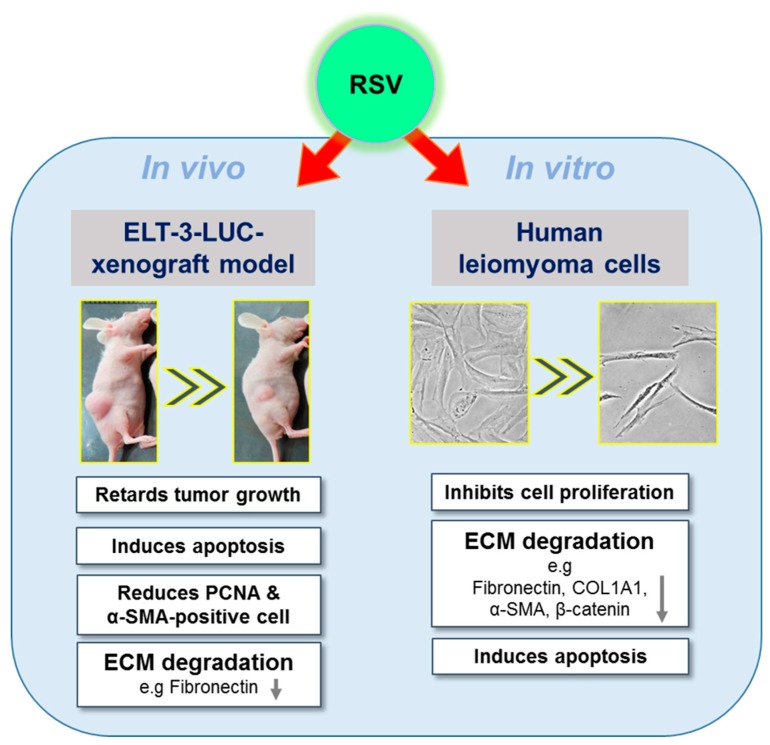
Schematic diagram of how the mechanism of RSV inhibits the growth of uterine fibroids. RSV significantly suppressed the tumor growth of ELT-3-LUC-xenografted mice and enhanced the Bax/Bcl-2 ratio, as well as reducing the proportion of PCNA and α-SMA-positive cells and the protein expression of fibronectin in an in vivo experiment. RSV also significantly inhibited the viability of primary human leiomyoma cells (magnification, ×200), induced apoptosis, and regulated the ECM-related gene (fibronectin) and proteins (COL1A1, vimentin, α-SMA, and β-catenin) in in vitro experiments. Abbreviations: RSV, resveratrol; ECM, extracellular matrix; PCNA, proliferating cell nuclear antigen; COL1A1, collagen type 1; α-SMA, alpha-smooth muscle actin.

The present study demonstrated that RSV suppressed tumor growth in vivo and inhibited primary human leiomyoma cells in vitro (Figure 6). In addition, RSV regulated ECM-associated protein expression. These findings indicate that RSV has the potential to reduce hyperplasia of leiomyoma cells. To the best of our knowledge, this is the first study to demonstrate the inhibitory potential of RSV on UF growth in vivo and may encourage further studies to highlight the molecular mechanisms involved in RSV and UF.

## Figures and Tables

**Table 1:** Sequences of quantitative reverse transcription–polymerase chain reaction (qRT–PCR) primers.

Gene	Forward (5′ to 3′)	Reverse (5′ to 3′)
*FN1* ^1^	GGCCAGTCCTACAACCAGTAT	TCGGGAATCTTCTCTGTCAGC
*GAPDH* ^2^	TGCACCACCAACTGCTTAGC	GGCATGGACTGTGGTCATGAG

^1^*FN1*, fibronectin; ^2^*GAPDH*, glyceraldehyde 3-phosphate dehydrogenase.

## Abbreviations

RSV	Resveratrol
ELT-3	Eker uterine leiomyoma cells
UF	Uterine fibroids
ECM	Extracellular matrix
PCNA	Proliferating cell nuclear antigen
α-SMA	Alpha-smooth muscle actin
SDS-PAGE	Sodium dodecyl sulfate polyacrylamide gel electrophoresis
IVIS	In vivo imaging system (IVIS)
